# The Spanish version of the reflective functioning questionnaire: Validity data in the general population and individuals with personality disorders

**DOI:** 10.1371/journal.pone.0274378

**Published:** 2023-04-06

**Authors:** Eduardo Ruiz-Parra, Guadalupe Manzano-García, Roberto Mediavilla, Beatriz Rodríguez-Vega, Guillermo Lahera, Ana I. Moreno-Pérez, Alberto M. Torres-Cantero, Juan Rodado-Martínez, Amaia Bilbao, Miguel Ángel González-Torres

**Affiliations:** 1 Department of Neurosciences, Faculty of Medicine and Nursing, University of the Basque Country, Bilbao, Spain; 2 Department of Psychiatry, Basurto University Hospital, Osakidetza Basque Health Service, Bilbao, Spain; 3 Biomedical Research Networking Centre in Mental Health (CIBERSAM), Madrid, Spain; 4 Department of Educational Sciences, University of La Rioja, Logroño, Spain; 5 Department of Psychiatry, School of Medicine, Autonomous University of Madrid (UAM), Madrid, Spain; 6 La Paz Hospital Institute for Health Research (IdiPAZ), Madrid, Spain; 7 Department of Psychiatry, Clinical Psychology and Mental Health, La Paz University Hospital, Madrid, Spain; 8 Department of Medicine and Medical Specialties, Faculty of Medicine and Health Sciences, University of Alcalá, Alcalá de Henares, Madrid, Spain; 9 Department of Psychiatry, Príncipe de Asturias University Hospital, Alcalá de Henares, Madrid, Spain; 10 Department of Public Health Sciences, School of Medicine, University of Murcia, Murcia, Spain; 11 Department of Preventive Medicine, Virgen de la Arrixaca University Clinical Hospital, El Palmar, Murcia, Spain; 12 Department of Psychiatry, School of Medicine, University of Murcia, Murcia, Spain; 13 Department of Psychiatry, Reina Sofía University Hospital, Murcia, Spain; 14 Research Unit, Basurto University Hospital, Osakidetza Basque Health Service, Bilbao, Bizkaia, Spain; 15 Health Service Research Network on Chronic Diseases (REDISSEC), Madrid, Spain; 16 Kronikgune Institute for Health Services Research, Barakaldo, Spain; Universität Kassel: Universitat Kassel, GERMANY

## Abstract

**Introduction:**

Mentalization or reflective functioning (RF) is the capacity to interpret oneself or the others in terms of internal mental states. Its failures have been linked to several mental disorders and interventions improving RF have a therapeutic effect. Mentalizing capacity of the parents influences the children’s attachment. The Reflective Functioning Questionnaire (RFQ-8) is a widely used tool for the assessment of RF. No instrument is available to assess general RF in Spanish-speaking samples. The aim of this study is to develop a Spanish version of the RFQ-8 and to evaluate its reliability and validity in the general population and in individuals with personality disorders.

**Methods:**

602 non-clinical and 41 personality disordered participants completed a Spanish translation of the RFQ and a battery of self-reported questionnaires assessing several RF related constructs (alexithymia, perspective taking, identity diffusion and mindfulness), psychopathology (general and specific) and interpersonal problems. Temporal stability was tested in a non-clinical sub-sample of 113 participants.

**Results:**

Exploratory and confirmatory factor analyses suggested a one-factor structure in the Spanish version of the RFQ-8. RFQ-8 understood as a single scale was tested, with low scorings reflecting genuine mentalizing, and high scorings uncertainty. The questionnaire showed good internal consistence in both samples and moderate temporal stability in non-clinical sample. RFQ correlated significantly with identity diffusion, alexithymia, and general psychopathology in both samples; and with mindfulness, perspective taking, and interpersonal problems in clinical sample. Mean values of the scale were significantly higher in the clinical group.

**Discussion:**

This study provides evidence that the Spanish version of the RFQ-8, understood as a single scale, has an adequate reliability and validity assessing failures in reflective functioning (i.e., hypomentalization) in general population and personality disorders.

## Introduction

Mentalization is the capacity to understand ourselves or the others in terms of mental states (e.g., feelings, wishes, goals, desires and attitudes) [1]. As a result thereof, behavior and emotional experiences become more meaningful and predictable, especially in the context of close and intimate relationships [2].

Several dimensions of mentalization have been described, including explicit or automatic versus implicit or controlled mentalization; self-focused versus other-focused mentalization; mentalization based on internal experience versus mentalization based on external cues; and cognitively versus affectively focused mentalization [2].

Genuine mentalization combines (a) the ability to form relatively accurate models of the mind, with (b) the awareness that any certainty is conditioned by the inevitable opacity of mental states [1]. On the basis of these two aspects, two types of failures in mentalization have been described: hypomentalization, reflecting excessive uncertainty about mental states; and hypermentalization, reflecting excessive certainty about mental states in the absence of appropriate evidence [1, 3].

Failures in mentalization have been linked to a vulnerability to various psychopathological conditions [4], and the interventions aimed at improving mentalization have proven to positively modify its course [5]. Furthermore, it has been suggested that the ability of the caregivers to mentalize in their relationship with the infant may determine the security of the child’s attachment [6].

Mentalization has been related to constructs such as theory of mind, mindreading, social cognition, metacognition, empathy, mindfulness, alexithymia, identity, and others. They are not completely equivalent concepts, but valid and easy-to-use measurement instruments are available for them. These instruments have been used as indirect measures of mentalization. However, they only measure some dimensions of mentalization and not others (e.g., measuring alexithymia with the well-known Toronto Alexithymia Scale can only indirectly assess oneself and emotional dimensions of mentalization), and depart from other theoretical premises, so their use is limited [7, 8].

Reflective function (RF) is an operationalization for research purposes of the mental capacities that generate mentalization [6]. In order to measure them, the Reflective Functioning Scale (RFS) [9], which evaluates RF on transcriptions of the Adult Attachment Interview (AAI) [10], was developed. This instrument constitutes the gold standard for RF measurement. Subsequently, other instruments were designed to measure RF and parental reflective functioning (PRF), focused on the representations that parents have about their own children, reproducing the narrative coding system of the RFS [11–16]. More recently, the Computerized Reflective Functioning (CRF) [17] has been developed. It identifies linguistic markers associated with high RF in narrative transcripts, and can be applied under induced psychological stressful situations [18], thereby facilitating the assessment of automatic mentalization [19].

As RF research progressed, it became apparent that narrative coding instruments were complex and required substantial resources in terms of training and time, resulting in difficulties in conducting large sample studies [20]. The need for easy-to-apply and easy-to-score questionnaires arose. Accordingly, some instruments have been developed: (a) the Mentalizing Stories for Adolescents (MSA) [21], assessing RF in adolescents; (b) the Parental Reflective Functioning Questionnaire (PRFQ) [22], assessing PRF; and, (c) assessing RF in adults, the Mentalization Questionnaire (MZQ) [23], the Mentalization Scale (MentS) [24], the Certainty About Mental States Questionnaire (CAMSQ) [25], the Multidimensional Mentalizing Questionnaire (MMQ) [26], and the 8-item Reflective Functioning Questionnaire (RFQ-8) [27], which also has a 6-item version [28, 29], a 15-item version (RFQ-15) [30], and a 18-item version (RFQ-18) [31]. Some modified versions of the RFQ have been developed for adolescents: the 8-item Reflective Functioning Questionnaire for Youth (RFQY) [4], the 23-item PFQY Scale B [32], and the 6-item RFQY Scale B [32]. The RFQ8 was developed by the group that coined the term mentalization from a more extensive questionnaire, the RFQ-54 [33], whose psychometric assessment data have not been published.

The RFQ-8 is a self-report questionnaire originally developed in English, which has been translated into several languages [34]. Its psychometric properties have been evaluated in general population in English, French, Italian, Greek, German, Persian and Polish; in individuals with personality disorder, and other psychiatric patients, in English, Italian, and German; and in adult type 1 diabetes population in Greek [4, 27–29, 35–38].

The RFQ-8, as originally conceived, contains two subscales, the certainty about mental states scale (RFQc) (items 1 to 6), and the uncertainty about mental states scale (RFQu) (items 2, 4, 5, 6, 7 and 8). High scorings of the scales are assumed to reflect respectively hypermentalization and hypomentalization [27].

In developing the RFQ, two types of items were designed in relation to their scoring system: some used a median-scoring method (extreme answers reflected lower scores, while responses reflecting genuine mentalization–i.e., less extreme answers–received the highest scores), and the others, the so called Scale B items [34], used a polar-scoring method (stronger agreement–or disagreement in case of inverted items–yielded higher RF scores). Only median-scored items were considered for further development of the questionnaire, but, as extreme answers for these items did not discriminate between hypermentalization and hypomentalization, the decision was made to convert them into polar-scored items, using a double-scoring system (scoring them in one direction to reflect hypomentalization, and in the opposite to reflect hypermentalization). For example, the response to the item “I always know what I feel” were rescored from 1, 2, 3, 3, 2, 1 to 1, 2, 3, 4, 5, 6 to account for certainty scale, and to 6, 5, 4, 3, 2, 1 to account for uncertainty scale. In order to capture more extreme levels of the variables, the items were then rescored as 1 = 0, 2 = 0, 3 = 0, 4 = 0, 5 = 1, 6 = 2 and 1 = 2, 2 = 1, 3 = 0, 4 = 0, 5 = 0, 6 = 0. Additionally, the initial 6-point Likert-type scale was changed to a 7-point Likert-type one, rescoring the items from 2, 1, 0, 0, 0, 0 to 3, 2, 1, 0, 0, 0, 0, and from 0, 0, 0, 0, 1, 2 to 0, 0, 0, 0, 1, 2, 3. The final 6 items of each scale of the RFQ-8 were selected by the criteria of showing the highest loadings on their respective factor across a series of exploratory and confirmatory factor analyses (CFA) [27]. Since items 2, 4, 5 and 6 belong to both scales, they are double-scored.

The RFQ-8 has been extensively used to assess RF in research aimed at (a) defining its dysfunction (usually hypomentalization) in groups of patients or other populations [39–47], and how it mediates symptomatic expression [35, 48–67]; (b) assessing change in psychotherapy [68–75]; and (c) understanding the relationship of RF to attachment and parenting [27, 60, 76–79]. Surprisingly, much of this research [27, 43–48, 50, 52, 53, 57, 59–68, 71, 77] suggest that higher RFQc scores may indicate more appropriate mentalization [51, 68], perhaps reflecting confidence in mental states as valid explanations for emotional experiences and behaviors [48].

Recently, during German validation studies [28, 29], some concerns have arisen about the double-scoring procedure, the factor structure of the RFQ, and, again, the validity of RFQc scale. Double-scoring causes problems in factor analysis. Given that only one rating is provided for four of the eight items on the 7-point scale, eight rescaled scores on RFQc and RFQu are mutually determined. Those pairs of scores are not independent and information overlaps. Nine of the 16 theoretical combinations for each of the two paired scores are mathematically impossible. This results in polychoric correlations between several scores approaching r = -1, clearly indicating that the double-scores are redundant [29]. The two-factor model, as proposed in initial validation studies [4, 27, 35] has been questioned arguing that a negative correlation between the two factors is artificially induced because the residual correlations of double-scored item pairs are restricted to zero [29]. A change in scoring procedure has been proposed, avoiding double-scoring and rescuing the scoring previously used in the RFQ design process (i.e., 1, 2, 3, 4, 5, 6, 7), except for item 7, reversely scored due to its content polarity with the other items [28, 29]. Additionally, evidence has being provided via exploratory factor analysis (EFA) and CFA that a one-factor model can sufficiently explain the observed covariation of the responses to RFQ-8 items when using this new way of scoring [28, 29, 38]. Finally, neither RFQ-8 certainty pole (using new proposed way of scoring), nor its certainty scale (using the previously proposed way of scoring) tend to show positive associations with negative outcomes (i.e., psychopathology) when looking for U shape [28, 29] or linear correlations [27, 38, 43–48, 50, 52, 53, 57, 59–68, 71, 77], thus suggesting the inability of the RFQ-8 to assess hypermentalization [29, 51]. In this sense, the CAMSQ [25] has been recently validated to fill the gap of specifically assess hypermentalization.

As no instruments assessing general RF in Spanish are available (only those assessing PRF), the need for reliable and valid instruments to measure RF in Spanish has been pointed out [8]. The RFQ-8, provided that the issues associated with its scoring system and factor structure are properly analyzed, could be a reasonable option to address this need, since it is an easy-to-use instrument, designed to measure mentalization difficulties, and has already been widely used in research.

The purpose of the present study is to develop a Spanish version of the RFQ-8 conceptually, semantically and operationally equivalent to the original version, and to assess its reliability and construct validity in two samples of Spanish general population and patients with personality disorder.

## Material and methods

### Participants

A non-clinical convenience sample drawn from general adult population was selected from different Spanish Autonomous Communities (Basque Country, La Rioja, Murcia, and Madrid). The sample was recruited by means of informative talks to pre and postgraduate students of a medical school and a business school, and, by word of mouth, among hospital and university staff, their families and friends. Inclusion criteria were to be over 18 years old, and to provide written informed consent. Exclusion criteria were showing general verbal or Spanish language communication difficulties, a clinical diagnosis of cognitive impairment, or having undergone psychiatric or psychological treatment on a mental health facility during the year prior to inclusion in the study. The final sample included 602 participants. A sub-sample of 113 participants was selected by convenience to evaluate retest reliability.

A clinical convenience sample (from Basque Country and Madrid) of 41 adult participants with a diagnosis of personality disorder assessed by means of the SCID-II interview [80] was selected in order to assess the psychometric properties of the questionnaire in a clinical sample.

The study protocol was approved by the Basque Country Clinical Research Ethics Committee.

### Translation of the questionnaire

The original English RFQ was translated into Spanish using a standard translation back-translation procedure aimed at ensuring conceptual, semantic and operational equivalence [81]. Translation was carried out by independent native Spanish and English translators [82]. Clarity, appropriateness and equivalence of the questionnaire were assessed by a group of experts in psychometric evaluation. In order to assess comprehension, a pilot study with 10 subjects with a maximum academic level of secondary education or equivalent (compulsory in Spain) was also included. The original and the Spanish versions of the questionnaire have been included with in S1 and S2 Appendices.

### Measurements

Sociodemographic data, and diagnostic standardization data in the clinical sample, were obtained at the university and clinical facilities linked to the study. Questionnaires were completed online at home in the non-clinical sample, and via paper and pencil, and on line at the mentioned facilities in the clinical sample. Retest were completed 2–3 weeks after first test.

The subjects completed a battery of questionnaires, which included:

The Spanish version of the 8-item **Reflective Functioning Questionnaire** (RFQ-8): each item of the questionnaire is scored on a 7-point Likert-type scale, ranging from "strongly disagree" to "strongly agree". Two different ways of scoring are used, (a) one reflecting the originally proposed for the two scales [27], where the RFQc items are rescored as 1 = 3, 2 = 2, 3 = 1, 4 = 0, 5 = 0, 6 = 0, 7 = 0; RFQu items are rescored as 1 = 0, 2 = 0, 3 = 0, 4 = 0, 5 = 1, 6 = 2, 7 = 3, except for item 7, which is rescored as 1 = 3, 2 = 2, 3 = 1, 4 = 0, 5 = 0, 6 = 0, 7 = 0; and each scale final score is the average of its item scores [34]; (b) and the other, reflecting a single scale [28, 29, 38], keeping all the items in the scoring previously used in the RFQ design process (scoring 1 to 7, and inversely rescoring for item 7). The scale final score is the average of all the items scores, with high values indicating uncertainty about mental states and, as previous research suggests [28, 29, 38], low values indicating genuine mentalization.

The **Perspective Taking** subscale of the **Interpersonal Reactivity Index** (IRI) [83]: the IRI is a 28-item self-report scale that assesses implicit empathy. It has been validated in the Spanish general population [84]. The IRI has four subscales: Perspective Taking (PT), Fantasy, Empathic Concern, and Interpersonal Stress. PT include 7 items, and indicates the spontaneous tendency to adopt the psychological point of view of the others (a cognitive dimension of empathy). Each item is scored on a 5-point scale anchored by 0 (A = “does not describe me well”) and 4 (D = “describes me very well”). A weak correlation (r = -0.18, p<0.05; r = -0.26, p<0.01) has been reported between PT and RFQu [27, 37]. Internal consistency of the scale in the present study in non-clinical sample (α = 0.82) and clinical sample(α = 0.92) was good.

The **Toronto Alexithymia Scale** (TAS-20) [85, 86] is a 20-item self-report scale which has been validated both in the Spanish general population and in psychosomatic patients in Spain [87]. The TAS-20 consists of a total alexithymia score (TASt), and three subscales: Difficulty Identifying Feelings (DIF), Difficulty Describing Feelings (DDF), and, less relevant for our study, Externally Oriented Thinking. Each item is evaluated according to a Likert-type scale from 1 to 6, ranging from "very much in disagreement" to "very much in agreement", and rescored to 0 to 5. Both scales of the RFQ showed moderate correlations with TASt (RFQc r = -0.53, p<0.05; RFQu r = 0.43, p<0.05), and DIF subscale (RFQc r = -0.45, p = 0.001; RFQu r = 0.57, p = 0.001; RFQc r = -0.56, p<0.01; RFQu r = 0.50, p<0.01) in previous validation studies [4, 35, 37]. Internal consistency in our non-clinical sample (TASt α = 0.87, DIF α = 0.87, DDF α = 0.85) and clinical sample (TASt α = 0.89, DIF α = 0.93, DDF α = 0.75) was good.

The Spanish version of the **Mindful Attention Awareness Scale** (MAAS) [88, 89] is a 15-item self-report scale that assesses an individual’s dispositional capacity to be attentive and aware of the experience of the present moment in daily life (mindfulness). The questionnaire has been validated in Spanish clinical and general populations [90]. It is scored with a Likert-type scale with a range from 1 ("almost always") to 6 ("almost never"). Weak correlations between MAAS and RFQu scale (r = -0.33, p<0.01; r = -0.24, p<0.01), and weak or no correlation with RFQc scale (r = 0.34, p<0.01; r = 0.08, p: n.s.) have been reported [27, 37]. Internal consistency in the present non-clinical sample (α = 0.90) and clinical sample (α = 0.92) was excellent.

The 83-item **Personality Organization Inventory** (IPO-83) [91]: It’s a 83-item self-report inventory, validated in Spanish general and clinical population [92], that evaluate the dimensions of the structural organization of the personality. The IPO-83 is composed of (a) three primary scales: Identity Diffusion (21 items), use of Primitive Defenses (16 items), and Reality Testing (20 items); and (b) two secondary scales: Aggression (18 items), and Moral Values (11 items, 3 of them are shared with the primary scales). The items are evaluated on a 5-point Likert scale, ranging from "never true" to "always true" [91]. The primary scales of a larger version, the IPO-136, showed moderate correlations with the RFQu in the original validation study (ID r = 0.57, p<0.01; PD = 0.52, p<0.01, RT = 0.54, p<0.01), but only ID scale moderately correlated with the RFQc (r = -0.41, p<0.01) [27]. The total scores of the IPO-16, a shorter version of the inventory–composed only of the primary scales–, used as indicators of severity of personality disfunction, correlated with the unidimensional RFQ-8 (r = 0.72, p<0.001; r = 0.64, p<0.05) [29]. Internal consistency of all the scales in the present non-clinical sample (α from 0.79 to 0.92), and clinical sample (α from 0.73 to 0.94) was good.

The **Symptom Checklist 90 Revised** (SCL-90-R) [93]: it’s a widely used 90-item self-report checklist, validated in Spanish general population [94, 95]. It assesses 9 symptom patterns scales (Somatization, Obsessive-compulsive, Interpersonal Sensitivity, Depression, Anxiety, Hostility, Phobic Anxiety, Paranoid Ideation, and Psychoticism scales) and 3 psychological discomfort indexes (Global Severity Index -GSI-, Positive Symptom Total -PST-, and Positive Symptom Distress Index -PSDI-). It is scored with a 5-point Likert-type scale with a range from 0 ("absence of the symptom") to 4 ("total presence of the symptom"). Psychological distress, as measured by a short version of the checklist, the SCL-10, moderately correlated with RFQc (r = -0.47, p<0.01) and RFQu (r = 0.59, p<0.01) in a sample of diabetic patients [36]. Internal consistency of all the scales in the present non-clinical sample (α from 0.80 to 0.98), and clinical sample (α from 0.86 to 0.99) was good.

The **Beck Depression Inventory-II** (BDI-II) [96]: it’s a 21-item self-report scale that assesses the presence and severity of depressive symptoms. It has been validated in Spanish general and clinical populations [97–99]. Items are scored in a 4-point Likert-type scale, ranging from total absence of the symptom to most severe presence of it; except for items 16 and 18 that scored in a 7-point scale, but are conveniently rescored to 0 to 4. Moderate correlations of the BDI-II with RFQu in clinical (r = 0.53, p<0.01) and non-clinical samples (r = 0.40, p<0.01) have been reported [27]. Internal consistency of the inventory in the present non-clinical sample (α = 0.92) and clinical sample (α = 0.93) was excellent.

The **Personality Inventory for DSM-5 Brief Form** (PID-5-BF) [100, 101]: it’s a 25-item self-report scale, validated in Spanish general and clinical populations [102], which evaluates 5 domains of personality traits (Negative Affect, Detachment, Antagonism, Disinhibition and Psychoticism), dysfunctional variants of the Big Five model [103]. Items are scored on a 4-point scale, ranging from 0 ("totally false or often false") to 3 ("very true or often true"). RFQ-8 showed moderate correlations with PID-5-BF total score (r = 0.67, p<0.001); and with Negative Affect (r = 0.60, p<0.001), Detachment (r = 0.46, p<0.001), Disinhibition (r = 0.44, p<0.001), and Psychoticism (r = 0.52, p<0.001) domains in one study [29]. Internal consistency of PID-5-BF total score in our non-clinical sample (α = 0.87) and clinical sample (α = 0.92) was good.

The PID-5-BF was designed as a tool to support the mixed categorical-dimensional alternative model of personality disorders in the DSM-5. This model requires assessment on a continuum of personal (identity and self-direction) and interpersonal (empathy and intimacy) functioning levels, and combines it with the assessment of 5 major personality domains [101]. In the present study, in order to assess the relationships between failures in mentalization and the different dimensions of personality, the instrument is used independently from the assessment of personality functioning.

The 32-item **Inventory of Interpersonal Problems** (IIP-32) [104]: it’s a self-report inventory that assesses interpersonal functioning, validated in Spanish general population and individuals with personality disorders [105]. It assesses interpersonal behaviors that the subject has difficulty carrying out or carries out excessively using a 5-point Likert-type scale, ranging from 0 ("not at all") to 4 ("extremely"). It provides an overall score, the one used in our study, and scores on eight scales that reflect different interpersonal dimensions. Using the IIP-62, a larger version of the inventory, weak correlations between a general measure of interpersonal problems and RFQc (r = -0.16, p<0.05), and RFQu (r = 0.32, p<0.01) have been reported [27]. The IIP-32 overall score moderately correlated with the RFQ-8 (r = 0.54, p<0.001) in another study [29]. Internal consistency of the overall score in the present non-clinical sample (α = 0.90) and clinical sample (α = 0.92) was excellent.

### Data analysis

Given the problems with the double-scoring procedure, which reproduce with our data (S1 Table); and the growing evidence towards a lack of specificity of the certainty scale, and a one-factor model, the decision was made to use the new way of scoring in the present validation study. Nevertheless, all analysis were performed using both ways of scoring. The results using the originally proposed way of scoring [27] can be found in the Supporting Information section.

The percentage of subjects scoring at the lowest possible level of the scale (floor effect) and the highest possible level (ceiling effect) were examined. Floor and ceiling effects should be minimal, and we used 15% as the critical value for those effects [106].

Construct validity: Factor analyses.

To study the structural validity of the questionnaire, replicating the methodology used in by Spitzer et al. [28] and Müller et al. [29], both confirmatory factor analysis (CFA) and exploratory factor analysis (EFA) were performed on the non-clinical sample using the new scoring system. CFA was used to investigate the hypothesized, recently proposed, one-factor structure [28, 29]; and the two-factor structure of the RFQ as proposed by the creators of the questionnaire [27]. Weighted least squares mean and variance adjusted (WLSMV) estimator was used. Different fit indices were evaluated using the following criteria: (a) chi squared divided by the degrees of freedom, the result of which had to be ≤3 to be acceptable; (b) the root mean squared error of approximation (RMSEA), where a value <0.08 was considered acceptable; and (c) the Tucker-Lewis Index (TLI) and the comparative fit index (CFI), both of which had to be >0.90 to be satisfactory [27, 107–110]. Factor loadings were also examined, and those ≥0.30 were considered acceptable [111]. Therefore, if the model surpassed these acceptability criteria, it was considered acceptable. The Lagrange multiplier test, which identifies paths or covariances that should possibly be added to the model to improve the fit was used when the model needed modification. For the comparison of the two-factor and one-factor models, as the models are non-nested, we used the Akaike information criterion (AIC) and the Bayesian information criterion (BIC), where lower values indicate that the model fits better [112]. Further, the two-factor EFA was performed considering the Promax oblique factor rotation. Factor loadings were also examined, and those ≥0.30 were considered acceptable [111].

Additionally, a CFA was performed using the originally proposed way of scoring [27], based on the creators’ recommendations [34], and using the same goodness-of-fit indices.

Reliability.

Internal consistency of the RFQ-8 subscales was assessed with Cronbach’s alpha coefficient [113]. A coefficient over 0.70 was considered acceptable [114]. The temporal stability was examined by performing a test–retest and calculating the intraclass correlation coefficient (ICC). Values from 0.50 to 0.75 pointed to moderate reliability, from 0.75 to 0.90 to good reliability, and values over 0.90 indicated excellent reliability [115, 116].

Construct validity: Convergent/discriminant and known-groups validity.

We assessed convergent and discriminant validity by analyzing the relationship between the RFQ-8 and identity diffusion, perspective taking, mindfulness, and alexithymia with Spearman correlation coefficients. We hypothesized a significant moderate to strong correlation (0.40 to 0.79 in absolute value) [117] between the RFQ-8 scale and the related constructs.

The relationship between the RFQ-8 domains and different measures of psychopathology (SCL-90, BID-II), personality domains (PID-5-BF) and interpersonal functioning (IIP-32) was assessed, again using the Spearman correlation coefficient.

We examined known-groups validity by comparing non-clinical with clinical group. We hypothesized that the comparation will significantly discriminate between both groups. For the comparison, the t-test was used, or the non-parametric Wilcoxon or Kruskal-Wallis test when normality was not met.

Effects were considered significant at *p*<0.05. Statistical analyses were performed with SAS^®^ for Windows statistical software, version 9.4 (SAS Institute, Inc., Carey, NC); and MPlus, version 6.1 [118].

## Results

### Demographics

Both the non-clinical and the clinical sample were predominantly females (respectively 82.56%, and 78.05%). Medium age of subjects was 24.28 years (SD = 10.32) in non-clinical sample and 40.44 years (SD = 10.66) in clinical sample. Educational level was higher for non-clinical sample, with only 2.16% with a maximum level of secondary school. As a large part of the sample was recruited among university students, most of the sample (74.42%) had a high school education. The educational level of the clinical sample was more balanced, with 34.5% of subjects with a maximum level of secondary education, and 29.25% with university education, the latter probably in relation to the age of the subjects

Neither ceiling effect, nor floor effect was noted for the questionnaire using the new scoring method, contrasting with a slight floor effect found for the RFQu scale (15.45% of the subjects scored at the lowest possible level of the scale) when using the original scoring method.

### Exploratory and confirmatory factor analyses

**Fig 1:**
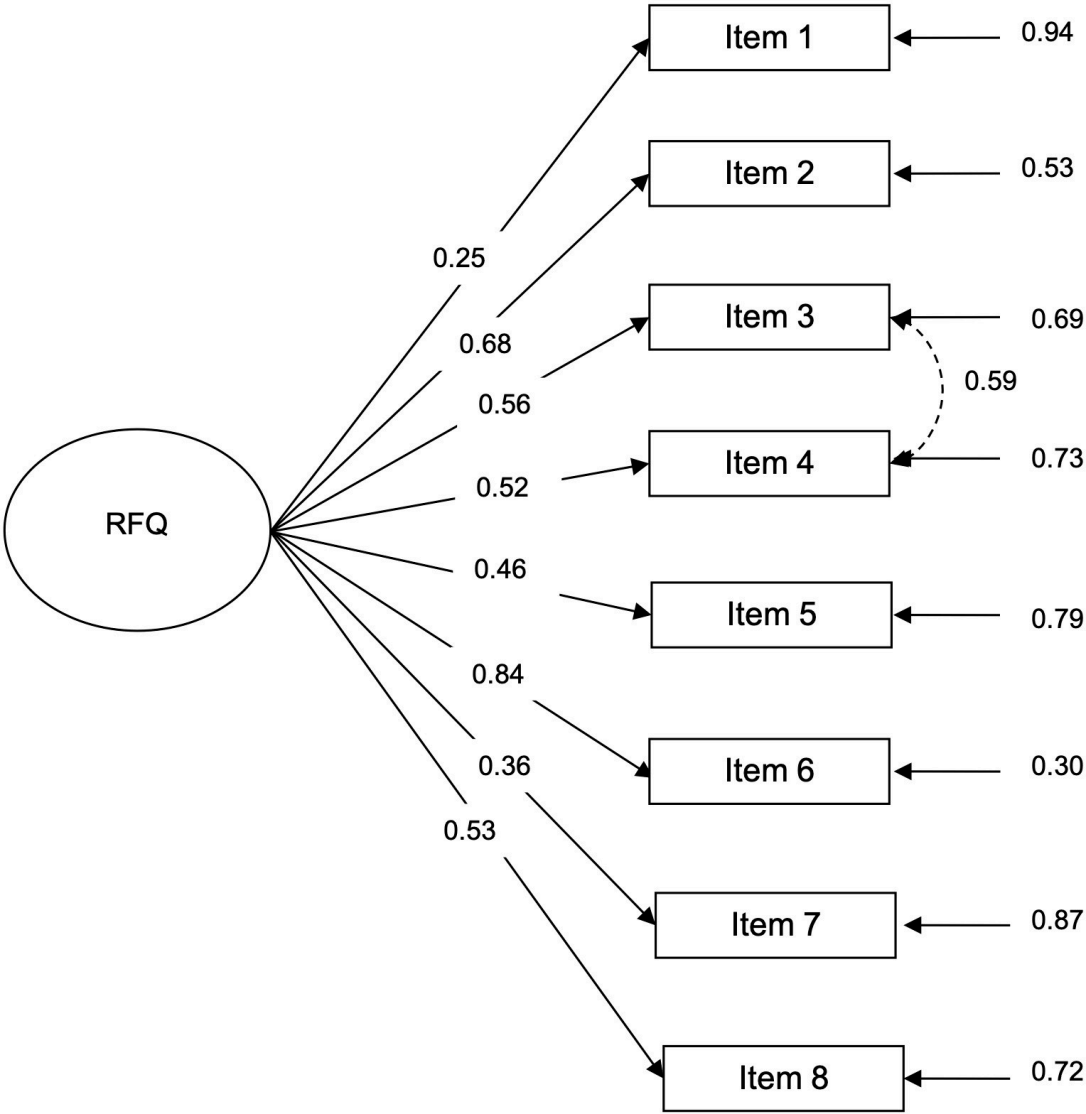
Confirmatory factor analysis for the one-factor structure in the non-clinical sample. Note: The standardized factor loadings and error variances are shown. As in Spitzer et al. [28], Müller et al. [29], and Wozniak-Prus et al. [38] studies, the error of items 3 and 4 were allowed to covariate.

**Fig 2:**
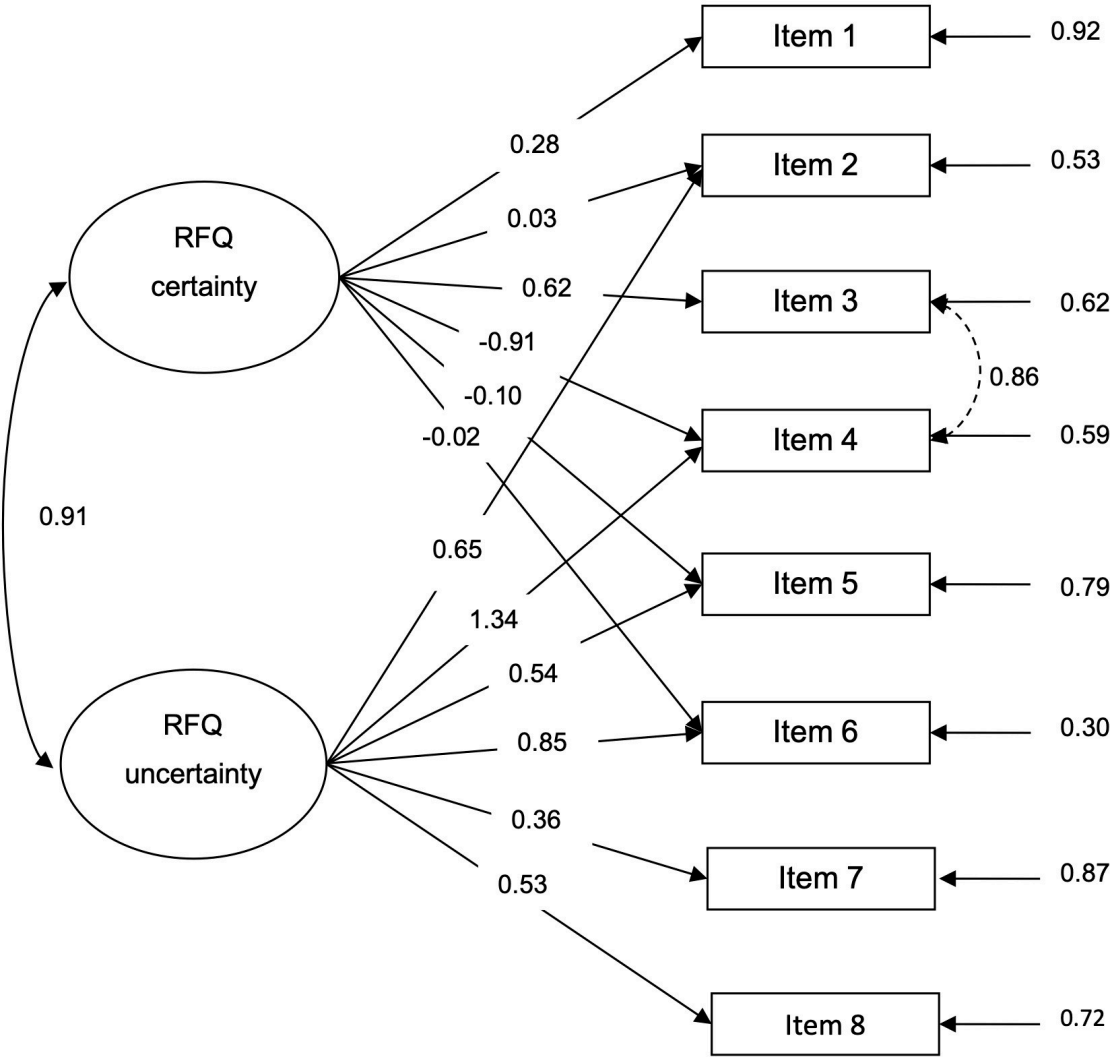
Confirmatory factor analysis for the two-factor structure in the non-clinical sample. Note: The standardized factor loadings, error variances and covariance among exogenous variables are shown. As in Spitzer et al. [28], and Müller et al [29] studies, the error of items 3 and 4 were allowed to covariate.

Results from the CFA for the one-factor structure showed satisfactory fit indices (Table 1), and all factor loadings were greater than 0.30, except for item 1, which was slightly lower (Fig 1). CFA testing a two-factor structure only partially fitted (Table 1), showing four items loading bellow 0.30 and one above 1 (Heywood case) (Fig 2). Further, the AICs and BIC were lower for the one-factor structure (Table 1), indicating that the one-factor structure fits better than the two-factor structure.

**Table 1:** CFA in non-clinical sample using a one-factor and a two-factor model: goodness-of-fit indices and comparison criterions.

	N	χ^2^	df	χ^2^/df	RMSEA (90%CI)	TLI	CFI	AIC	BIC	ABIC
**RFQ 1f**	602	53.46	19	2.81	0.055 (0.038–0.073)	0.96	0.97	17954.02	18064.02	17984.65
**RFQ 2f**	602	45.73	14	3.27	0.061 (0.042–0.082)	0.95	0.97	17956.28	18088.28	17993.05

Note: RFQ 1f: One-factor model; RFQ 2f: two-factor model; χ^2^: Chi Square; df: Degrees of freedom; RMSEA: Root Mean Square Error of Approximation; CI: Confidence Interval; TLI: Tucker-Lewis Index; CFI: Comparative Fit Index; AIC: Akaike Information Criterion; BCI: Bayesian Information Criterion; ABIC: Adjusted BIC.

**Fig 3:**
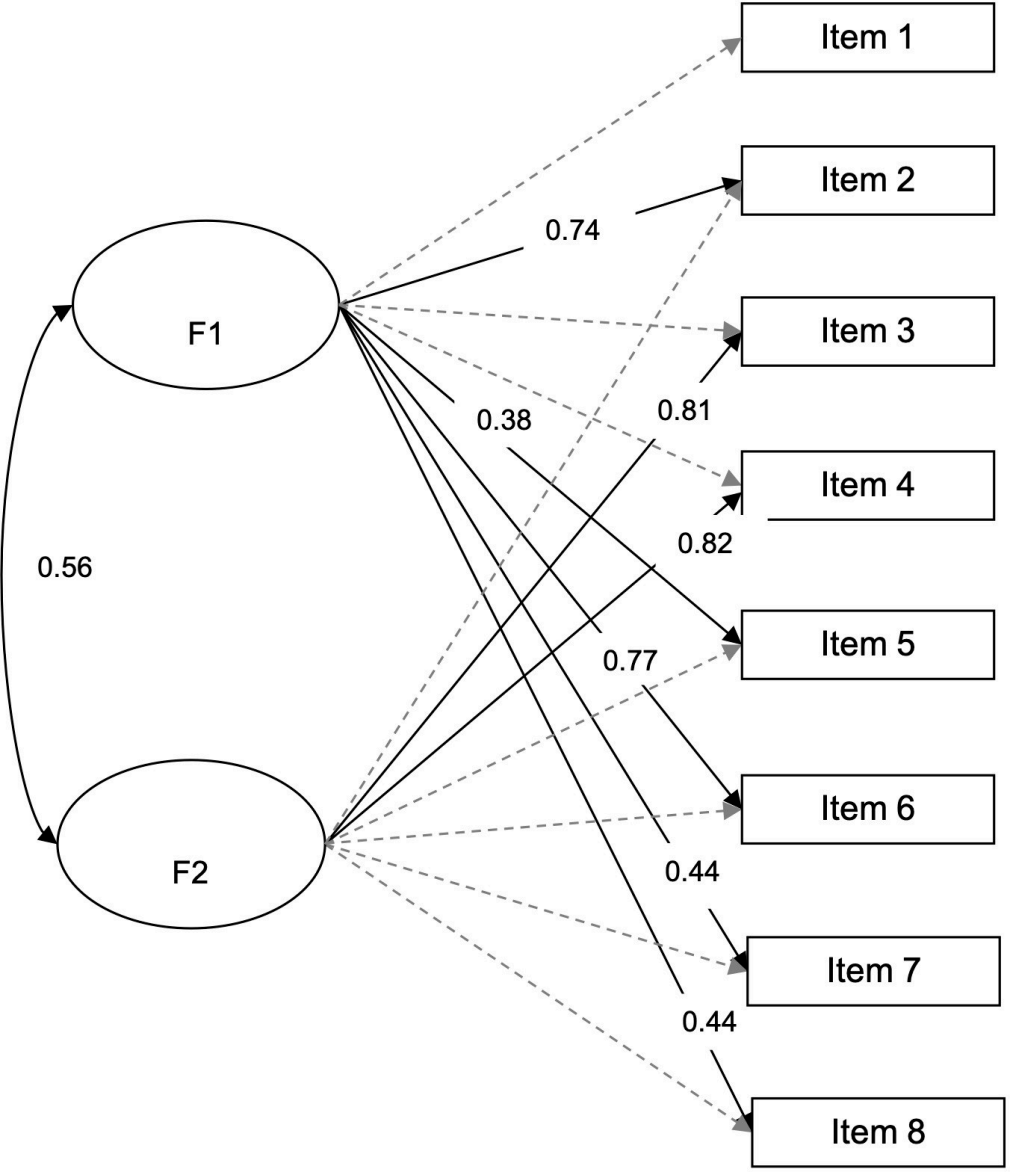
Exploratory factor analysis for the two-factor structure in the non-clinical sample using Promax rotation. Note: The factor loadings are shown, and those smaller than 0.30 are grayed out. The percentage of variance explained by the two factors was 53.01%.

The results of the two-factor EFA (Fig 3) were far from the original two-factor structure (with items 2, 5, 6, 7, and 8 belonging to one factor; items 3, and 4 to another; and item 1 loading below 0.30).

### Reliability

The questionnaire showed a good internal consistency, with alpha values above 0.7 in both the non-clinical and clinical sample. Test-retest reliability evaluation with a subsample of the non-clinical sample showed moderate reliability for the RFQ-8 (Table 2).

**Table 2:** Reliability indices (internal consistency and temporal stability) for non-clinical and clinical groups.

	Cronbach’s Alpha	Test-Retest ICC
**Non-clinical sample**	0.763	0.746*
**Clinical sample**	0.783	-

*p<0.001

Only removing the item 1 seems to improve internal consistency, but it does it in such a way (α improved to 0.779 in non-clinical sample, and to 0.847 in clinical sample) that the decision was made to test convergent and know-groups validity maintaining all the items, without removing low loading items, and those that overlap in content, as it was made in studies using the RFQ-6 [28, 29]

### Convergent validity

**Mentalizing-related constructs.** In the non-clinical sample, RFQ-8 correlated moderately positively with the IPO-83 Identity Diffusion scale and with the Total TAS-20 scores. On the other hand, it showed a weak negative correlation with the MAAS and with the IRI Perspective Taking scale (Table 3).

**Table 3:** Correlations between RFQ and measures of mentalizing-related constructs among non-clinical and clinical sample.

			IPO		MAAS+	PR_IRI+	TAS			
			ID+	PD			T+	DIF		DDF
**RFQ**										
**Non-clinical**										
		n	258		254	323	323			
		rho	0.581*	0.496*	-0.286*	- 0.223*	0.487*	0.565*	0.296*	
**Clinical**										
		n	41		41	41	41			
		rho	0.817*	0.692*	-0.448**	- 0.424**	0.685*	0.770*	0.413***	

Note 1: IPO: Inventory of Personality Organization; ID: Identity Diffusion Scale; PD: Primitive Defenses Scale; MAAS: Mindful Attention Awareness Scale; PT_IRI: Perspective Taking Scale of Interpersonal Reactivity Index; TAS: Toronto Alexithymia Scale; T: Total; DIF: Difficulty Identifying Feelings Subscale; DDF: Difficulty Describing Feelings Subscale; +: Hypothetical Constructs to test; rho: Spearman Correlation Coefficient.

Note 2:

* p< 0.001;

**p < 0.01

In the clinical sample, the RFQ-8 showed a moderate to strong correlation in the expected direction with all the hypothetical mentalizing-related constructs (Table 3).

Correlation with psychopathology measures.

The RF-8 showed significant correlations with severity of general psychopathology, as measured by the Global Severity Index of the SCL-90, both in the clinical and in the non-clinical sample. It also correlated with each of the specific symptom scale in the clinical sample, and with many of them (except somatization, depression, hostility and phobic anxiety; but with absolute values of the correlation close to 0.4) in the non-clinical sample. On the other hand, the RFQ-8 showed a moderate correlation with the severity of depression, measured by the BDI-II, but only in the clinical sample (Table 4).

**Table 4:** Correlations between RFQ-8 and measures of psychopathology among non-clinical and clinical sample.

			RFQ			
			Non-clinical		Clinical	
			n	rho	n	Rho
**SCL-90-R**			293		40	
	Somatization			0.391*		0.457***
	Obsessive-Compulsive			0.416*		0.590*
	Interpersonal Sensitivity			0.409*		0.512**
	Depression			0.390*		0.485***
	Anxiety			0.423*		0.542**
	Anger-Hostility			0.361*		0.578*
	Phobic Anxiety			0.356*		0.542**
	Paranoid Thought			0.406*		0.585*
	Psychoticism			0.377*		0.564**
	Global Severity Index+			0.442*		0.548**
	Positive Symptoms Total			0.424*		0.491***
**BDI-II**+			300	0.330*	40	0.408**
**PID-5-BF**			311			
		PID5 Overall+		0.477*		
		Negative Affect		0.484*		
		Detachment		0.235*		
		Antagonism		0.253*		
		Disinhibition		0.416*		
		Psychoticism		0.354*		
**IIP-32**			293		40	
	IIP Overall+			0.382*		0.450***

Note 1: SCL-90-R: Symptom Checklist 90 Revised; BDI-II: Beck Depression Inventory II; PID-5-BF: Personality Inventory for DSM 5 Brief Form; IIP-32: Inventory of Interpersonal Problems 32. +: Hypothesized correlations; rho: Spearman Correlation Coefficients.

Note 2:

*p< 0.0001;

**p < 0.001;

***p< 0.01

The RFQ-8 correlated with the severity of personality dysfunction in the non-clinical sample. Finally, the RF-8 correlated with general interpersonal difficulties in the clinical sample (Table 4).

Group differences.

RFQ-8 mean values also discriminated between the non-clinical group and the clinical group of individuals with personality disorder (Table 5).

**Table 5:** Group differences between the non-clinical and the clinical samples.

			RFQ-8		
		N	Mean	SD	p value
**Non-clinical**		602	3.86	1.09	
**Personality disorders**		41	4.74	1.32	
					<0.0001

## Discussion and conclusions

### Translation and equivalence

According to the thorough translation procedure, the resulting version of the questionnaire apparently shows an adequate conceptual, semantic and operational equivalence with the original questionnaire. However, the new version inherits from the original some issues related to face and content validity, previously pointed out by Müller et al. [29]. The RFQ-8 includes only one question assessing thinking about other people; and seven questions regarding oneself, including five about feelings and two about thinking about behaviors. Items 2 and 6 are equivalent in content [27]. The RFQ-8 shows a loss of content validity with respect to its previous version, the RFQ-54.

Additionally, all items (except item 7) make statements about a state of uncertainty, resulting in a loss of face validity of the RFQc scale when applying the original scoring method, and, probably, in a lack of capacity of the RFQ-8 to measure a hypermentalization pole when applying the new scoring method [29]. Maybe the RFQ-8 could have more adequately represented the complexity of the RF definition if its items had not been selected from the RFQ-54 only by purely mathematical criteria.

### Characteristics of the samples

The non-clinical sample has a large sample size [111], providing sufficient robustness for the factor analyses, and other statistical analyses; and the clinical sample size is suitable for the analysis of convergent and known-groups validity. The overrepresentation of the female population in both samples might cause problems in the application of the questionnaires to a more homogeneous population. However, there is no previous evidence of significant differences in RF between both sexes [36]. In the non-clinical sample, a young population, with a medium-high educational level, is overrepresented, which would require an evaluation of the performance of the scales when applying the RFQ-8 to a more diverse population. The clinical sample differs significantly from the non-clinical sample in terms of age (higher in the former), and educational level (more diverse in the former). These differences should be considered when interpreting known-groups validity.

### Floor and ceiling effects

The absence of ceiling and floor effects when using the new scoring method is not surprising, and may be indicative of the capacity of the questionnaire to discriminate a wider range of mentalization difficulties (i.e., hypomentalization). Furthermore, it makes sense that the analysis revealed a slight floor effect for the RFQu scale, especially when applying the RFQ-8 in the nonclinical population, since this scale is expected to better capture severe mentalization deficits [27]. Original rescoring system was designed to capture extremes.

### Factorial structure of the questionnaire

Replicating the results of more recent studies [28, 29, 38], our study provides consistent evidence that a one-factor model adequately explains the observed covariation of RFQ-8 responses when using the new scoring system (proposed to evaluate concerns about the double-scoring procedure, the factor structure of the RFQ, and the validity of the RFQc scale [29]), challenging the capacity of the RFQ-8 to measure two different mentalization failures. In contrast with other validation studies [4, 27, 35–37] using the original scoring method, goodness-of-fit indices suggest that the RFQ-8 fails to adjust to a two-factor model when using CFA applying both the new and the original scoring methods (S1 Fig).

In our study, when one-factor CFA is performed using the new scoring system, item 1 is the only one that exhibits a factor loading bellow 0.30 (0.25), reproducing one study evaluating unidimensional RFQ [38], but not others [28, 29]. It may be worth noting that this is the only item that addresses mentalization about the others [29].

In Spitzer et al study [28] item 7 showed a negligible factor loading (0.14), and in Müller et al study [29] showed the lowest factor loadings in clinical sample (0.34) and both non-clinical samples (0.45, and 0.49). Besides, the authors proposed that items 3 and 4 largely overlap in their content (so their errors were allowed to covariate in both CFA). In those studies, the decision was made to remove items 4 and 7 [28, 29] to produce the RFQ-6. Although in Müller et al. study [29] all analyses were performed using both the 6-item and the 8-item versions leading to similar results. In our study the factor analyses made by the previous authors were replicated, including the decision to allow error correlations between items 3 and 4, as was suggested by the results of Lagrange multiplier test. It should be considered that the original authors suggested to limit the number of possible error correlations to a minimum, allowing only error correlations between items similar in formulation or meaning, but they explicitly cited items 2 and 6 (without excluding other possibilities) as overlapping in content [27]. In our study, only in the originally proposed two-dimensional CFA using double-scoring, error correlations between items 2 and 6, and between items 3 and 4 were allowed (S1 Fig).

Maintaining a more conservative position than some previous authors [28], we decided to keep all the items of the RFQ-8. Although the internal consistency of the questionnaire improved only if item 1 was removed, it did it in a scarcely relevant way, and the advantages of keeping a unified questionnaire in different languages are clear.

### Reliability

The results of the present study demonstrate that the Spanish version of the RFQ8 is a reliable instrument, with a good internal consistency and acceptable temporal stability. This replicates the findings of previous validation studies using the new scoring method [28, 29, 38].

### Convergent validity

One of the inevitable limitations of the study is that criterion validation is not possible, as the RFS, the gold standard in the measure of mentalization, is not available in Spanish. Besides, due to the lack of availability of other specific mentalizing self-reported questionnaires (like the MZQ, the MentS, the CAMSQ, or the MMQ) in Spanish, convergent validity relies on comparing the RFQ with less specific related constructs, as has been usual in previous validation studies.

The hypothesis of the RFQ-8 as correlating with the degree of identity diffusion both in the nonclinical and in the clinical samples is confirmed. The results are consistent with previous quantitative studies linking hypomentalization with identity diffusion and conditions where identity diffusion predominates [27, 57]. Such studies conceive hypomentalization as a causal or mediating variable. Nevertheless, mentalizing capacity and identity are probably related to each other in several ways. The achievement of mentalizing capacity enables the establishment of a sense of identity [119]. Moreover, mentalizing and identity disturbances are intertwined in personality disordered patients [120]. Finally, exploring identity demands thinking in terms of mental states regarding oneself and, according to the object relations model underlying the IPO-83 [121], regarding the others.

The RFQ-8 is also correlated with the Primitive Defenses scale of the IPO-83. Such correlation was somewhat expected, given the closely related nature of identity diffusion and primitive defenses in the model underpinning the IPO-83, as reflected in its original validation study factor analysis [91]. Some primitive defense mechanisms, such as splitting, involve inaccessibility at one point to a substantial part of internal experience about oneself or the others, thereby making difficult to understand oneself or the others in terms of elaborate and complex intentional mental states. As expected, the correlations of the RFQ-8 with identity diffusion and primitive defenses were higher in the personality disordered patients sample.

Replicating previous research [4, 35, 37], this study confirms the hypothesis of a correlation between hypomentalization and alexithymia, as reflected by the total alexithymia scores. Alexithymia might be conceived as an indirect measure of lack of emotional mentalization about oneself. The content of the RFQ-8 is biased toward these two dimensions of mentalizing, since 5 out of its 8 items assess the ability to mentalize feelings or emotions about oneself. As expected, the correlations were higher in the clinical sample. Additionally, the RFQ-8 specifically correlated with the Difficulty in Identifying Feelings subscale, the scale that most accurately reflects the reported dimensions of mentalization. The Difficulty in Expressing Feelings subscale of the TAS-20 assesses a general inhibited behavior when expressing feelings, hence its lower correlation with the RFQ-8 is understandable.

In previous studies using Perspective Taking scale [27, 37], only a weak to very weak correlation with hypomentalization (measured by RFQu scale) was found in a non-clinical sample. In a recent study conducted on a non-clinical sample in the US, Müller et al. [29] found unidimensional RFQ-8 correlated poorly with measures of mentalizing others, warning about the possibility that the instrument may not adequately measure mentalization regarding the others. In our study, a moderate negative correlation was found in the clinical sample, but in the non-clinical sample the correlation was weak, although stronger than in the original validation study [27]. These inconsistent correlations result in the RFQ-8’s ability to assess other-focused dimension of mentalization remains controversial. Perspective taking denotes a conscious effort to put oneself in the other’s position and adopt his or her point of view. In this regard, mentalizing about the other is underrepresented in the RFQ-8, as only item 1 accounts for it, which could explain weaker correlations than initially expected.

The same pattern of correlations reproduces with the measure of mindfulness: a moderate negative correlation with RFQ-8 in clinical sample, and a weak correlation in non-clinical sample. Previous studies using MAAS or other scales measuring mindfulness showed week correlations of the construct with hypomentalization in non-clinical samples. All these findings could be accounted for by differing emphases between the mentalization and mindfulness approaches. Mindfulness, unlike mentalizing, defines a perceptual and non-evaluative activity, not only encompassing mental states, and strictly focused on the present moment.

The study confirms that the RFQ-8 shows a strong correlation with several measures of psychopathology, either global or specific. The correlation is particularly strong when applying the questionnaire to personality-disordered subjects. In this population there is not only an association between hypomentalization and severity of general psychopathology (measured by the Global Severity Index of the SCL-90-R), and severity of depression (measured by the BDI-II), but also, a correlation is appreciated with each of the specific psychopathology measurement scales of the SCL-90-R, that cover a wide variety of symptomatic dimensions, including depression, anxiety, phobias, obsessive-compulsiveness, somatization, hostility, paranoid ideation, feelings of inferiority and inadequacy, and psychoticism.

In the nonclinical sample, the RFQ-8 correlated adequately with the overall severity of personality dysfunction and with the negative affect and disinhibition domains of the PID-5-BF. This study confirms the correlation between hypomentalization and personality difficulties found in previous studies, which mainly showed moderate to strong correlations with uncertainty scale [4, 27] or unidimensional RFQ [29, 38]. Only one study [35] that used a clinician-rated questionnaire to measure personality disfunction in individuals with borderline personality disorder showed a weak correlation with uncertainty scale.

The correlation of the RFQ-8 with negative affectivity and disinhibition in our study replicates previous studies [29, 38], and it is consistent with the assumption that inadequate mentalizing capacity (i.e., hypomentalization) produces emotional dysregulation and impulsivity [1]. However, some authors have advised that several RFQ-8 items directly assess impulsive behaviors in the context of emotional lability that may be the result of difficulties in mentalization, but may have other causes. This may produce an artificial inflation of correlations between the RFQ and indicators of personality pathology [29]. A communality analysis by Müller et al demonstrated that, although the RFQ-8 reflected impairments in mentalizing, 30% of the observed associations between the RFQ-8 and indicators of personality dysfunction were due to variance shared with measures of emotional lability and impulsivity [29]. Furthermore, an item-level analysis suggested that items 3, 4, 5, and 8 converged with measures of emotional lability and impulsivity rather than with measures of mentalization, and thus those items may be responsible for the artificially inflated correlations [29]. These findings should be considered when interpreting the associations of the RFQ-8, understood as a measure of mentalization, with other constructs, given that the instrument could also be measuring a combination of impulsivity and emotional dysregulation that is partly independent of mentalization difficulties, and that could act as a confounding factor.

The correlation between hypomentalization and the presence of interpersonal problems was moderate in the clinical sample, in agreement with previous findings using the IIP-32 [29]. The correlation was significant but weak when tested in a non-clinical sample for the first time. Some authors have suggested that hypomentization predicts interpersonal problems in individuals with personality disorder, but do it only indirectly, via emotional dysregulation and impulsivity [51].

Consistent with the hypothesis that personality disorders are mainly based on failures of mentalization [122], the group of individuals with personality disorders exhibit significantly higher mean scores of the questionnaire than the non-clinical group.

In the absence of measures in our language that directly measure mentalization, our study, in addition to assessing the fairly well-established relationship between hypomentalization and psychopathology, advances in the sense of comparing the questionnaire with mentalization related constructs, such as those used in the initial validation studies [4, 27, 32]. These constructs are now evaluated using the RFQ-8 as a one-factor questionnaire using the new scoring method. Some of them showed adequate correlations in the clinical sample with measures of cognitive empathy, besides mentalization about the others underrepresentation in the RFQ-8; and mindfulness, besides mentioned differing emphases in the approaches. Furthermore, we have understood alexithymia, and specially its DIF scale, as one of this closed and more genuine mentalization related constructs, measuring lack of emotional mentalizing about oneself (and not only as a measure of psychopathology). In a similar way, normal identity exploration is not possible without the capacity of genuinely mentalizing about oneself and the others. It is therefore not surprising that both constructs correlate well with the RFQ in both clinical and non-clinical samples and must be considered beyond their role as psychopathological indicators.

The generalized presence of significantly stronger correlations in the clinical sample than in the non-clinical sample for all the constructs under study suggests a higher reliability and validity of the questionnaire in the group of patients with personality disorders, and points to the need for validation studies with larger samples for this group.

#### RFQ-8 as an off-line measure

The RFQ8 is an offline measure of mentalization. It requests the subject to reflexively assess his or her own general mentalizing ability, without ensuring a current interpersonal, affectively relevant context for the assessment, and without assuring a minimum level of stress to promote the emergence of mentalization failures. The RFQ-8 has a limited capacity to include the dynamic and contextual elements of mentalization [7]. It provides us with a global picture of the subject’s mentalizing ability focused on one of its deficits, hypomentalization. The use of a standardized stress-inducing tasks [18] enables the assessment of automatic mentalization with other instruments [19], but probably this is not the case wiht the RFQ-8. Nevertheless, it is an easy-to-use instrument that provides a direct measure of hypomentalization. Yet, the need to validate measures in Spanish of the other mentalization deficit, hypermentalization, remains. In response to growing evidence, original developers of RFQ are in the process of validating a new version of the RFQ with a hypermentalizing scale to replace the certainty scale. We are awaiting their and others findings with this measure.

## Conclusions

In summary, the Spanish version of the RFQ-8 is an off-line self-report reliable instrument with an adequate construct validity. The present study suggest a one-factor structure of the questionnaire. Using the new scoring method proposed, the RFQ-8, as an unidimensional questionnaire aimed at measure hypomentalization, correlates with several measures of psychopathology in clinical and non-clinical population; and with diverse mentalization related constructs, with stronger correlations in clinical sample. Design of a new scale to measure hypermentalization, with adequate face validity, and free of double-scoring problems is encouraged. Further research is needed in our community with measures of hypomentalization and hypermentalization with larger samples of individuals with personality disorders, and another psychopathology.

## Supporting information

S1 Appendix The reflective functioning questionnaire. (PDF)

S2 Appendix Spanish version of the RFQ-8. (PDF)

S1 Fig Originally proposed two-dimensional CFA model using double-scoring in non-clinical sample. (PDF)

S1 Table Polychoric correlations among items using the original scoring method. (PDF)

S2 Table Reliability indices (internal consistency and temporal stability), using double-scoring, for non-clinical and clinical groups. (PDF)

S3 Table Group differences between the non-clinical and the clinical samples, using double-scoring. (PDF)

S4 Table Correlations between RFQc and RFQu and measures of mentalizing-related constructs among non-clinical and clinical sample. (PDF)

S5 Table Correlations between RFQc and RFQu and measures of psychopathology among non-clinical and clinical sample. (PDF)

S1 Dataset Data used in the study. (XLSX)
