# Supplementary material for: The Spanish version of the reflective functioning questionnaire: Validity data in the general population and individuals with personality disorders
Source: PLoS One. 2023 Apr 6;18(4):e0274378. doi: 10.1371/journal.pone.0274378 (PMC10079014; doi:10.1371/journal.pone.0274378)
Supplement: S1 Appendix — (PDF) [file pone.0274378.s001.pdf]

S1 Appendix. The Reflective Functioning Questionnaire

Please work through the next 8 statements. For each statement, choose a number between 1 and 7 to say how much you disagree or agree with the statement, and write it beside the statement. Do not think too much about it – your initial responses are usually the best. Thank you.

Use the following scale from 1 to 7:

Strongly disagree	1	2	3	4	5	6	7	Strongly agree

1. People’s thoughts are a mystery to me.
2. I don’t always know why I do what I do.
3. When I get angry I say things without really knowing why I am saying them.
4. When I get angry I say things that I later regret.
5. If I feel insecure I can behave in ways that put others’ backs up.
6. Sometimes I do things without really knowing why.
7. I always know what I feel.
8. Strong feelings often cloud my thinking **.**
