# Supplementary material for: The Spanish version of the reflective functioning questionnaire: Validity data in the general population and individuals with personality disorders
Source: PLoS One. 2023 Apr 6;18(4):e0274378. doi: 10.1371/journal.pone.0274378 (PMC10079014; doi:10.1371/journal.pone.0274378)
Supplement: S2 Appendix — (PDF) [file pone.0274378.s002.pdf]

S2 Appendix. Spanish version of the RFQ-8

A continuación puede ver una serie de frases. Lea cada una de ellas y decida si está de acuerdo o en desacuerdo y hasta qué punto. Utilice la siguiente escala de valoración, puntuando 7 si está completamente de acuerdo y 1 si está en completo desacuerdo. El punto intermedio, si usted se siente neutral al respecto o indeciso, es 4.

	Muy en desacuerdo			⟷			Muy de acuerdo
1. Los pensamientos de la gente son un misterio para mí.	1	2	3	4	5	6	7
2. No siempre sé por qué hago lo que hago.	1	2	3	4	5	6	7
3. Cuando me enfado digo cosas sin saber realmente por qué las digo.	1	2	3	4	5	6	7
4. Cuando me enfado digo cosas de las que luego me arrepiento.	1	2	3	4	5	6	7
5. Si me siento inseguro puedo comportarme de una manera que molesta a los demás.	1	2	3	4	5	6	7
6. A veces hago cosas sin saber exactamente por qué.	1	2	3	4	5	6	7
7. Siempre sé lo que siento.	1	2	3	4	5	6	7
8. A menudo sentimientos muy fuertes nublan mi pensamiento.	1	2	3	4	5	6	7
