# Supplementary material for: The Spanish version of the reflective functioning questionnaire: Validity data in the general population and individuals with personality disorders
Source: PLoS One. 2023 Apr 6;18(4):e0274378. doi: 10.1371/journal.pone.0274378 (PMC10079014; doi:10.1371/journal.pone.0274378)
Supplement: S1 Fig — (PDF) [file pone.0274378.s003.pdf]

S1 Fig. Originally proposed two-dimensional CFA model using double-scoring in non-clinical sample.

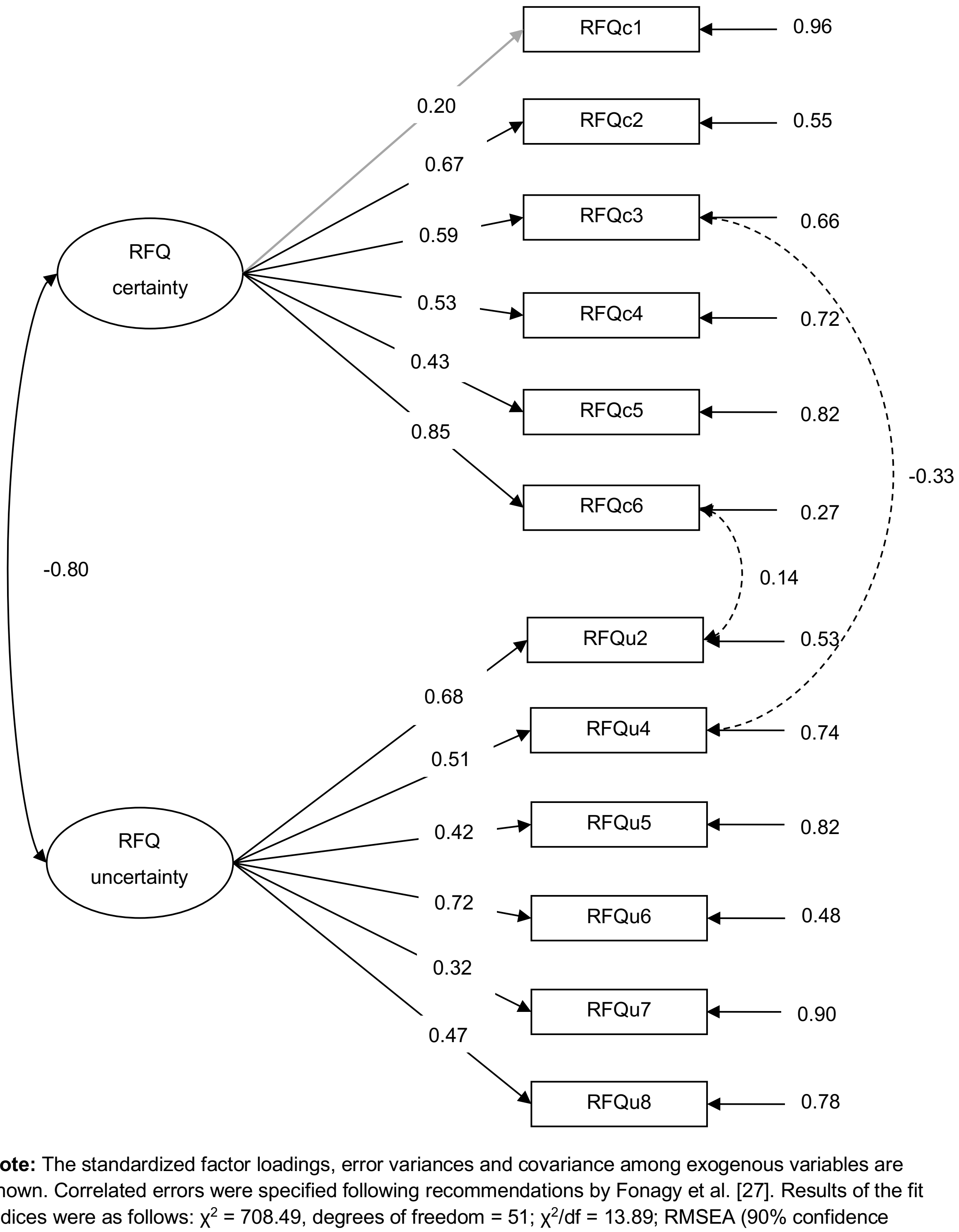

Note: The standardized factor loadings, error variances and covariance among exogenous variables are shown. Correlated errors were specified following recommendations by Fonagy et al. [27]. Results of the fit indices were as follows: χ^2 = 708.49, degrees of freedom = 51; χ^2/df = 13.89; RMSEA (90% confidence interval) = 0.146 (0.137 – 0.156); CFI = 0.70 and TLI = 0.61; AIC = 18878.56; BIC = 19050.17; and Adjusted BIC = 18926.36.
