# Supplementary material for: The Spanish version of the reflective functioning questionnaire: Validity data in the general population and individuals with personality disorders
Source: PLoS One. 2023 Apr 6;18(4):e0274378. doi: 10.1371/journal.pone.0274378 (PMC10079014; doi:10.1371/journal.pone.0274378)
Supplement: S1 Table — (PDF) [file pone.0274378.s004.pdf]

S1 Table. Polychoric correlations among items using the original scoring method.

	Certainty						Uncertainty					
**Certainty**	**RFQ8c1**	**RFQ8c2**	**RFQ8c3**	**RFQ8c4**	**RFQ8c5**	**RFQ8c6**	**RFQ8u2**	**RFQ8u4**	**RFQ8u5**	**RFQ8u6**	**RFQ8u7**	**RFQ8u8**
**RFQ8c1**	1											
**RFQ8c2**	0.1756	1										
**RFQ8c3**	0.2036	0.4829	1									
**RFQ8c4**	0.0950	0.4489	0.8022	1								
**RFQ8c5**	0.1057	0.3587	0.3419	0.3630	1							
**RFQ8c6**	0.1837	0.6980	0.6061	0.5523	0.4762	1						
**Uncertainty**												
**RFQ8u2**	-0.1001	-0.9983	-0.3828	-0.3238	-0.2856	-0.6129	1					
**RFQ8u4**	-0.1094	-0.3619	-0.6734	-0.9984	-0.2638	-0.4341	0.3443	1				
**RFQ8u5**	0.0029	-0.2661	-0.3013	-0.2528	-0.9991	-0.3848	0.3856	0.3598	1			
**RFQ8u6**	-0.1589	-0.5980	-0.4452	-0.3803	-0.3823	-0.9981	0.6597	0.4490	0.4354	1		
**RFQ8u7**	-0.1178	-0.4056	-0.1250	-0.1236	-0.1692	-0.2815	0.3703	0.1576	0.2556	0.3142	1	
**RFQ8u8**	-0.1315	-0.3123	-0.3078	-0.2503	-0.3513	-0.3946	0.4501	0.3665	0.5216	0.4854	0.2697	1
