# Supplementary material for: The Spanish version of the reflective functioning questionnaire: Validity data in the general population and individuals with personality disorders
Source: PLoS One. 2023 Apr 6;18(4):e0274378. doi: 10.1371/journal.pone.0274378 (PMC10079014; doi:10.1371/journal.pone.0274378)
Supplement: S2 Table — (PDF) [file pone.0274378.s005.pdf]

S2 Table. Reliability indices (internal consistency and temporal stability), using double-scoring, for non-clinical and clinical groups.

		Cronbach's Alpha	Test-Retest ICC
**Non-clinical sample**	RFQc	0.737	0.784*
	RFQu	0.736	0.628*
**Clinical sample**	RFQc	0.741	-
	RFQu	0.803	-

*p<0.001
