# Supplementary material for: The Spanish version of the reflective functioning questionnaire: Validity data in the general population and individuals with personality disorders
Source: PLoS One. 2023 Apr 6;18(4):e0274378. doi: 10.1371/journal.pone.0274378 (PMC10079014; doi:10.1371/journal.pone.0274378)
Supplement: S3 Table — (PDF) [file pone.0274378.s006.pdf]

S3 Table. Group differences between the non-clinical and the clinical samples, using double-scoring.

		RFQc			RFQu		
	N	Mean	SD	p value	Mean	SD	p value
**Non-clinical**	602	0.85	0.68		0.68	0.59	
**Personality disorders**	41	0.63	0.73		1.36	0.89	
				0.0106			<0.0001
