# Supplementary material for: The Spanish version of the reflective functioning questionnaire: Validity data in the general population and individuals with personality disorders
Source: PLoS One. 2023 Apr 6;18(4):e0274378. doi: 10.1371/journal.pone.0274378 (PMC10079014; doi:10.1371/journal.pone.0274378)
Supplement: S4 Table — (PDF) [file pone.0274378.s007.pdf]

S4 Table. Correlations between RFQc and RFQu and measures of mentalizing-related constructs among non-clinical and clinical sample.

	RFQc				RFQu			
	Non-clinical		Clinical		Non-clinical		Clinical	
	n	rho	n	rho	n	rho	n	rho
**IPO**	258		41		258		41	
ID+		-0.450***		-0.703***		0.580***		0.739***
PD		-0.406***		-0.599***		0.492***		0.625***
**MAAS**+	254	0.256***	41	0.576***	254	-0.259***	41	-0.370*
**PR_IRI**+	323	0.260***	41	0.422**	323	-0.154**	41	-0.417**
**TAS**	323		41		323		41	
T+		-0.423***		-0.683***		0.433***		0.626***
DIF		-0.490***		-0.754***		0.509***		0.699***
DDF		-0.201***		-0.430**		0.303***		0.442**

Note 1: IPO: Inventory of Personality Organization; ID: Identity Diffusion Scale; PD: Primitive Defenses Scale; MAAS: Mindful Attention Awareness Scale; PT_IRI: Perspective Taking Scale of Interpersonal Reactivity Index; TAS: Toronto Alexithymia Scale; T: Total; DIF: Difficulty Identifying Feelings Subscale; DDF: Difficulty Describing Feelings Subscale; +: Hypothetical Constructs to test; rho: Spearman Correlation Coefficient.

Note 2: *p< 0.01; **p < 0.01; ***p< 0.001
