# Supplementary material for: The Spanish version of the reflective functioning questionnaire: Validity data in the general population and individuals with personality disorders
Source: PLoS One. 2023 Apr 6;18(4):e0274378. doi: 10.1371/journal.pone.0274378 (PMC10079014; doi:10.1371/journal.pone.0274378)
Supplement: S5 Table — (PDF) [file pone.0274378.s008.pdf]

S5 Table. Correlations between RFQc and RFQu and measures of psychopathology among non-clinical and clinical sample.

	RFQc				RFQu			
	Non-clinical		Clinical		Non-clinical		Clinical	
	n	rho	n	rho	n	rho	n	rho
**SCL90R**	293		40		293		40	
Som		-0.317***		-0.225		0.390***		0.530***
Obs		-0.315***		-0.327*		0.442***		0.681***
IS		-0.316***		-0.289		0.446***		0.575***
Dep		-0.300***		-0.258		0.435***		0.606***
Anx		-0.316***		-0.331*		0.449***		0.665***
Hos		-0.322***		-0.439**		0.355***		0.575***
Pho		-0.284***		-0.303		0.358***		0.590***
Par		-0.333***		-0.344*		0.445***		0.652***
PsycS		-0.283***		-0.302		0.431***		0.620***
GSI+		-0.341***		-0.293		0.474***		0.646***
PST		-0.352***		-0.294		0.433***		0.538***
**BDI-II**+	300	-0.215***	40	-0.318*	300	0.391***	40	0.457**
**PID5BF**	311				311			
PID5O+		-0.411***				0.439***		
NA		-0.413***				0.4426***		
**IIP-32**	293		40		293		40	
IIPO+		-0.308***		-0.514***		0.387***		0.390**

Note 1: SCL90: Symptom Checklist 90; Som: Somatization Scale; Obs: Obsessive-Compulsive Scale; IS: Interpersonal Sensitivity Scale; Dep: Depression Scale; Anx: Anxiety Scale; Hos: Anger-Hostility Scale; Pho: Phobic Anxiety Scale; Par: Paranoid Thought Scale; PsychS: Psychoticism Scale; GSI: Global Severity Index; PST: Positive Symptoms Total; BDI-II: Beck Depression Inventory II ; PID5BF: Personality Inventory for DSM 5 Brief Form; PID5O: Overall; NA: Negative Affect Domain; IIP-32: Inventory of Interpersonal Problems 32; IIPO: Overall. +: Hypothesized correlations; ; rho: Spearman Correlation Coefficients.

Note 2: *p< 0.01; **p < 0.01; ***p< 0.00
